# A Novel Bisquaternary Ammonium Compound as an Anion Sensor—ESI-MS and Fluorescence Study

**DOI:** 10.3390/ijms25063467

**Published:** 2024-03-19

**Authors:** Marta Kowalska, Robert Wieczorek, Paula Gawryszewska, Remigiusz Bąchor

**Affiliations:** Faculty of Chemistry, University of Wroclaw, F. Joliot-Curie 14, 50-383 Wroclaw, Poland; marta.kowalska@uwr.edu.pl (M.K.); robert.wieczorek@uwr.edu.pl (R.W.); paula.gawryszewska-wilczynska@uwr.edu.pl (P.G.)

**Keywords:** ESI-MS, anion sensor, fluorescence, quaternary ammonium salts, TFA anion, computational analysis

## Abstract

Electrospray ionization mass spectrometry (ESI-MS) analysis is frequently associated with noncovalent adduct formation, both in positive and negative modes. Anion binding and sensing by mass spectrometry, notably more challenging compared to cation binding, will have major research potential with the development of appropriate sensors. Here, we demonstrated identification of stable bisquaternary dication adducts with trifluoroacetate (TFA^−^), Cl^−^ and HSO_4_^−^ in positive-mode ESI-MS analysis. The observed adducts were stable in MS/MS mode, leading to the formation of characteristic fragment ions containing a covalently bound anion, which requires bond reorganization. This phenomenon was confirmed by computational methods. Furthermore, given that anion detection and anion sensor chemistry have gained significant prominence in chemistry, we conducted an analysis of the fluorescent properties of bisquaternary ammonium compound as a potential anion sensor.

## 1. Introduction

A frequent phenomenon during electrospray ionization-mass spectrometry (ESI-MS) analysis is the observation of adduct formation in both the positive, which is more common, and the negative modes of ESI [1]. Commonly observed are sodium, potassium, ammonium, lithium or silver adducts, depending on the Lewis basicity of the analyzed compound [2]. These adducts may arise as a result of the presence of sodium and potassium ions in glassware, solvent contamination or additives. The formation of the mentioned adducts is possible because the surface excess charge of the ESI nanodroplet can be carried by various types of cations [3]. It was found that various metal adducts may be formed in ESI-MS depending on the nature of the analyzed compound, type of solvent and ESI source parameters [4]. Usually, for these adducts, no fragmentation apart from cation loss is observed. For example, carbohydrates may have fragmentation that differs significantly from the fragmentation of protonated ions, which provides useful information about the structure of these compounds [2].

Apart from adducts with cations, it was confirmed that there is a possibility to form adducts with, for example, chloride anions during ESI-MS analysis in the negative ion mode of compounds such as glycolipids [5] and glycerophosphocholine lipids [6]. In addition, a method has been developed to enable MS analysis of analytes that do not have acidic sites and therefore show low-intensity [M-H]^−^ signals. The method was based on the addition of Cl^−^ ions present in solutions of chlorinated solvents, such as chloroform. Chloride ion attachment has been observed, for example, for aniline. In addition, for analytes with acidity lower than HCl, it was observed during fragmentation analysis (MSMS) to promote the formation of the [M-H]^−^ ion and other daughter ions, which consequently provided information about the structure of the compound [7].

Trifluoroacetic acid (TFA) is a commonly used additive for liquid chromatography–mass spectrometry (LC–MS) and high-performance liquid chromatography (HPLC) for peptides [8], proteins [9] and small basic molecules [10] due to its ability to reduce the silanol group effect from columns, which enhances the chromatographic peak shape [11]. However, when electrospray ionization is used in MS analysis, trifluoroacetic acid may cause ion suppression due to its ability to form gas-phase ion pairs with positively charged analyte ions (adduct formation), which reduces the signal intensity in the MS spectrum [12,13]. A reduction of 30% to 600% of signals has been observed in the case of small basic molecules [11]. 

Additionally, the ionization efficiency of certain compounds during the ESI-MS experiment is low, and its reliable identification of trace amounts is restricted. The application of fixed charge tags such as quaternary ammonium (QAT) [14], phosphonium [15] and sulphonium [16] salts is one of the most important approaches for increasing ionization efficiency and detection sensitivity. Recently, we developed a quaternary ammonium fixed charge tag for sensitive peptide detection by the LC–ESI-MS/MS method. Using nano-LC-ESI-MRM analysis, the subfemtomolar sensitivity of detecting peptides derivatized with the designed charge tag was estimated. Meanwhile, for protonated peptides, pico- and femtomolar levels were reached. The use of QAT reduces the limitations of the ESI-MS/MS analysis by enabling the analysis of compounds with low ionization efficiency or small amounts of compounds [17].

Pyrylium salts are well-known aromatic compounds containing a constant positive charge. Pyrylium salts have a positively charged oxygen atom, which shows high reactivity towards nucleophiles. Therefore, they have found application in the synthesis of pyridinium derivatives [18]. The constant positive charge of the pyridinium group increases ionization efficiency, which results in improved sensitivity for peptide detection. During MS/MS analysis, it was observed that TPP salt-modified peptides generated the abundant protonated 2,4,6-triphenylpyridinium ion. This fragment can serve as a reporter ion in Multiple Reaction Monitoring analysis [18]. The proposed solution was successfully applied by us in the development of a method of human podocin tryptic peptide identification in urine sediment hydrolysates using mass spectrometry and chemical modification in the form of charge derivatization [19]. We also investigated the possibility of noncovalent dimer formation of a quaternary ammonium denatonium cation in the gas phase in ESI-MS analysis as a consequence of amide hydrogen deprotonation [20].

An area of research that has recently been extensively studied is the chemistry of anion receptors. This field of study is concerned with the design of molecules that recognize, react with or sense compounds with a negative charge in their structure [21]. This topic is very attractive due to the wide range of applications of anion receptors. Anion receptors have been used in bioimaging, materials science as well as in caching and extracting toxic anions from industrial waste to prevent environmental pollution [22] and as potential drugs for diseases such as cystic fibrosis, which is associated with problems with chloride transport caused by faulty ion channels in epithelial cell membranes [21].

Most of the anion receptors use the hydrogen bonding interactions of the N-H⋯anion to bind the anions strongly. Therefore, the groups of compounds that have been widely used as receptors are pyrroles [23], indoles [24], ureas [25], amides [26] and squaramides [27], as well as 1,2,3-triazole-based anions (C–H hydrogen bond donors). Other groups of anion receptors include halogen bonding [28], chalcogen bonding [29] and anion–π interactions [30]. Pyridine and pyridine heterocycles have also been shown to be effective as anion receptors, which was widely described by Kilah and Beer [31].

Since anion detection has become an important area of chemistry, the fluorescent properties of potential anion sensors with various proposed anion recognition mechanisms have been extensively studied, especially in recent times [32]. 

The widely reported sensors are hydrogen-bond-based chemosensors [16]. An example is 1-hydroxycarbazole. It was shown that this sensor was able to recognize fluoride and chloride anions with high sensitivity by creating hydrogen bonding via hydrogen atoms in the pyrrolic nitrogen and hydroxy group. An increase in the fluorescence emission intensity of the sensor was observed with increasing amount of added anion [33]. Another group of anion sensors is halogen-bond-based chemosensors due to the possibility of halogen bond formation. Additionally, several boron-based chemosensors have been developed, such as derivatives containing a pinacol boronate group or chemosensor based on two pyrenylboronic acid derivatives. Many metal-based chemosensors, such as Fe-, Pt-, Ir-, Ru- and lanthanide-based chemosensors, were also developed. Another group of anion sensors are charged chemosensors, for instance, turn-on fluorescent Cl^−^ sensors from *Gloeobacter violaceu* or coumarin-connected carboxylic indolium near-infrared (NIR) turn-off charged chemosensors sensitive to CN^−^ anions. Another type of developed chemosensors are excimers (pyrene-labelled sequence-controlled polymers and fluorescent tetramide chemosensors for HP_2_O_7_^3−^). Different groups of chemosensors that can be distinguished are chemosensors based on emissive charge-transfer states promoted by anion–π interactions, for instance, dibromo-substituted dicationic pyrometallic diimide, as well as photoswitchable chemosensors and chemodosimeters [16]. The formation of specific and non-specific noncovalent molecular associates is characteristic of ESI-MS analysis of biomolecules. Understanding the interaction between two associated molecules is of significance not only from a biological point of view but also regarding gas phase analysis by mass spectrometry. 

As already mentioned, compounds containing quaternary nitrogen atoms can be used to increase the ionization efficiency and improve the detection capabilities of weakly ionizing compounds during ESI-MS analysis. Additionally, the formation of precursor ions in ESI-MS^2^ regarding mono- and bisquaternary ammonium compounds has been demonstrated [34]. In general, it is believed that the formation of adducts is responsible for the suppression of ionization (for example, the adduct with TFA during peptides analysis). However, it has been shown that the formation of adducts can increase the signal intensity for anionic species [35].

The ESI, EI, MALDI and DART mass spectrometry techniques have been used to characterize different disubstituted 4,4′-bipyridinium salts in order to determine which technique is best for determining these kinds of salts. It has been demonstrated that ESI-MS is more convenient for quantitative analysis, while MALDI and DART are suitable for qualitative analysis of proposed bisquaternary salts [36].

Numerous studies have also focused on determining the properties of policationic species and their interactions with various anions, including examining detection limits. It has been demonstrated that anions containing halogen atoms are detected with greater sensitivity than analogues without halogen (chaotropy of the anion). Furthermore, anions with a higher oxidation state have a lower detection limit. It has been demonstrated that dication compounds (ammonium dication, phosphorus dication, and ammonium–phosphorus dication) possess the ability to from adducts with various anions, thereby facilitating their detection in positive mode with different efficiency. The binding of these molecules is significantly strengthened as they pass from the solution phase to the gas phase. Surface tension studies have additionally shown that, during the formation of adducts, a surfactant in the ESI drop is formed, resulting in a significant increase in sensitivity [19].

Moreover, the possibility of forming adducts of bisquaternary ammonium cation (methylimidazole derivative) with organic and inorganic anions has also been analyzed. The analyzed ammonium dication formed adducts with 32 anions in the positive mode. The present approach provides very low detection limits for a variety of anions, especially bromochloroacetic acid, dichloroacetic acid and nitrate anion [37]. The results of the research conducted so far are extremely promising and encourage further research using ammonium dications.

While in ESI-MS analysis formation of TFA adduct in the gas phase is a common phenomenon [8], to the best of our knowledge, observation of TFA^−^, Cl^−^ and HSO_4_^−^ adducts during ESI-MS/MS analysis has not been reported. In the present work, we described the gas phase formation of noncovalent trifluoroacetic anion-bisquaternary ammonium adduct, chloride anion-bisquaternary ammonium adduct and sulfate anion-bisquaternary ammonium adduct, which were stable even under MS/MS analysis. The stability and geometry of the observed adducts were analyzed by quantum mechanical calculations using density functional theory (DFT). Furthermore, in the present work, we described the absorption and fluorescence properties of the obtained adducts; likewise, we described the possibility of using bisquaternary ammonium compound as a receptor for trifluoroacetate anions.

## 2. Results and Discussion

### 2.1. Mass Spectrometry Analysis

**Figure 1:**
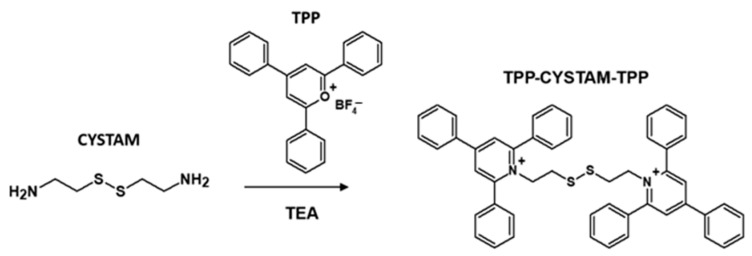
Schematic presentation of the synthesis of (TPP)2-CYSTAM. TEA-*N*,*N*,*N*-triethylamine.

The aim of this research was to synthesize a bisquaternary ammonium compound and to analyze the possibility of noncovalent trifluoroacetic anion-bisquaternary ammonium adduct, chloride anion-bisquaternary ammonium adduct and sulfate anion-bisquaternary ammonium adduct formation in the gas phase during ESI-MS analysis. The model bisquaternary ammonium dication in the form of 2,2′-disulfanediylbis(2,4,6-triphenylpyridinium) ((TPP)2-CYSTAM) was obtained in the reaction between cystamine and 2,4,6-triphenylpyrylium tetrafluoroborate under basic conditions (Figure 1), according to the method described in the Materials and Methods section. 

**Figure 2:**
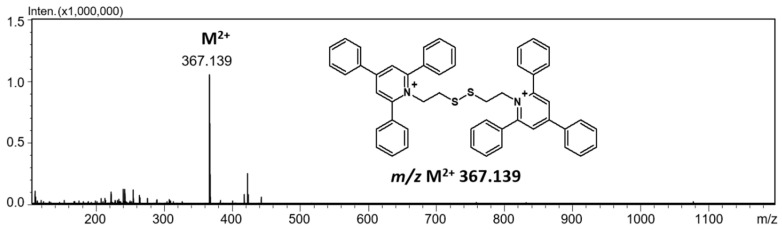
ESI-MS spectrum of (TPP)2-CYSTAM in positive mode. *m*/*z* range from 100 to 1200.

**Figure 3:**
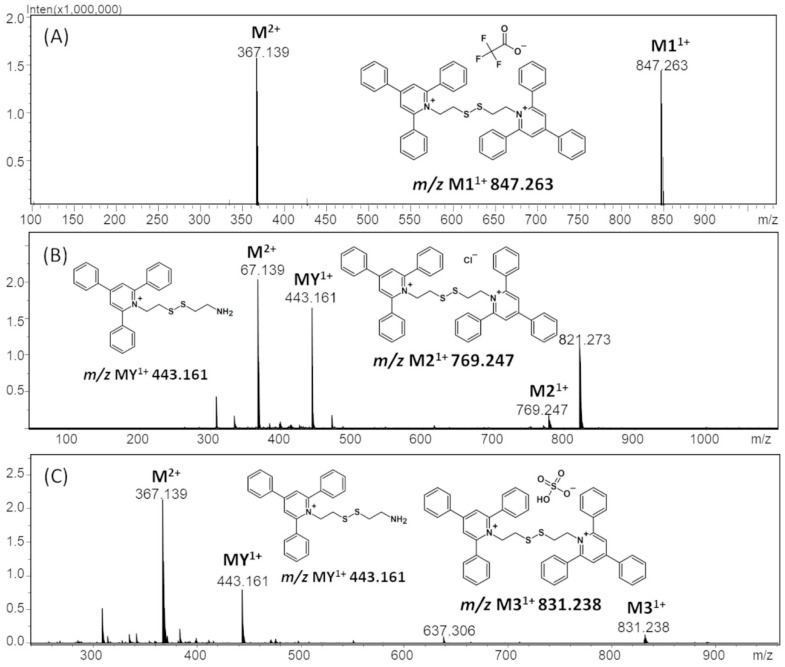
The ESI-MS spectra of (**A**) TFA-(TPP)2-CYSTAM adduct, (**B**) Cl-(TPP)2-CYSTAM and (**C**) HSO_4_-(TPP)2-CYSTAM in positive mode. *m*/*z* range from (**A**) 100 to 1000; (**B**,**C**) from 200 to 1000.

The mass spectrometry analysis of a bisquaternary ammonium dication in positive ion mode revealed the presence of the signal characterized ion at *m*/*z* 367.143, which corresponds to the M^2+^ ion (Figure 2). The synthesized compound was purified using the RP HPLC technique, where 1% TFA was used as a mobile phase additive. As a result, the following mass spectrum was obtained (Figure 3A). 

The obtained ESI-MS spectrum of (TPP)2-CYSTAM dication after HPLC analysis (Figure 3A) revealed the presence of signals at *m*/*z* 367.143 and 847.270. The detailed analysis showed that the signal at *m*/*z* 847.270 characterizes +1 ion containing two sulfur atoms and practically the same isotope pattern as in the case of the signal at *m*/*z* 367.143. Additionally, the mass of the formed ion was found to be 113 Da higher, which is characteristic of trifluoroacetate (112.986 Da). It is known that TFA used in the mobile phase can cause suppression effects and decrease signal intensity [12]. However, the synthesized compound has two positive charges, and the formation of TFA-(TPP)2-CYSTAM adduct neutralizes only one of the positive charges, which results in the formation of singly charged noncovalent adduct, stable in the gas phase. Based on this result, we decided to test the possibility of other anion bindings, including Cl^−^ and HSO_4_^−^, by the obtained dication. Analogously, ESI-MS spectrum of (TPP)2-CYSTAM in the presence of chloride anions Figure 3B) revealed the presence of, among others, signals at *m*/*z* 367.143, 443.161 and 769.247. These signals correspond to the following ions: (TPP)2-CYSTAM, TPP-CYSTAM and chloride adduct with (TPP)2-CYSTAM. The ESI-MS spectrum of HSO_4_-(TPP)2-CYSTAM adduct (Figure 3C) revealed the presence of mainly three signals at *m*/*z* 367.143, 443.161 and 831.238. Signals at *m*/*z* 367.143, 443.161 were also observed before (Figure 3B) and correspond to (TPP)2-CYSTAM and TPP-CYSTAM. The third signal at *m*/*z* 831.238 corresponds to the HSO_4_-(TPP)2-CYSTAM adduct. As in the case of TFA adduct, formation of Cl-(TPP)2-CYSTAM and HSO_4_-(TPP)2-CYSTAM adducts neutralizes only one of the positive charges of the bisquaternary ammonium compound, which results in the formation of stable singly charged noncovalent adduct in the gas phase. The presence of additional signals in the MS spectrum results from the fact that observations of the formation of adducts with Cl^−^ and HSO_4_^−^ anions were carried out on unpurified ammonium dication. The signal at *m*/*z* 443.161 characterizes the one-side-modified cystamine ion, which is a byproduct of the performed synthesis of the bisquaternary ammonium cation. The signal at *m*/*z* 821.273 is characterized by undefined impurity. 

Generally, no fragmentation besides cation or anion loss is observed in the case of the formed adducts in ESI-MS/MS. Therefore, to check the stability of the identified adduct, ESI-MS/MS analysis was performed (Figure S1).

On the obtained MS/MS spectra, one of the most characteristic is the signal at *m*/*z* 308.143 (Figure S1A–C), corresponding to the protonated form of 2,4,6-triphenylpyridine, which was previously described as a reporter ion generated during the fragmentation of TPP-derivatized peptide [18]. The second part of the molecule that remained after dissociation contains the positively charged quaternary nitrogen atom, which should be neutralized by the presence of a TFA^−^ (Figure S1A), Cl^−^ (Figure S1B) or HSO_4_^−^ (Figure S1C) anion if the formed adduct is stable in the gas phase. However, the obtained mass spectra present other signals that correspond to charged fragment ions with a higher mass than those resulting from (TPP)2-CYSTAM molecule fragmentation.

The fragmentation of the parent ion of TFA-(TPP)2-CYSTAM adduct at *m*/*z* 847.263 (M1^+^) revealed the formation of fragment ions [M1a]^+^, [M1b]^+^ and [M1c]^+^ at *m*/*z,* respectively, 540.127, 480.124 and 436.152 (Figure S1A). The detailed analysis of these signals revealed that these are singly charged TFA adducts. Fragmentation of parent ion of Cl-(TPP)2-CYSTAM adduct at *m*/*z* 769.245 (M2^+^) revealed the formation of fragment ions [M2a]^+^ and [M2b]^+^ at *m*/*z,* respectively, 462.111 and 402.108 (Figure S1B). Structures of these ions are analogous for [M1a]^+^ and [M1b]^+^ ions; the difference is the presence of the chloride anion instead of the trifluoroacetate anion. Fragmentation of the parent ion for HSO_4_-(TPP)2-CYSTAM at *m*/*z* 831.238 (M3^+^) revealed the formation of the fragment ions [M3a]^+^ and [M3b]^+^ at *m*/*z*, respectively, 524.102 and 464.098 (Figure S1, Panel C). The structures of these ions are analogous to those of [M1a]^+^, [M1b]^+^, [M2a]^+^ and [M2b]^+^ ions. Similarly, regarding adducts with TFA^−^ and Cl^−^, the detailed analysis showed that these signals correspond to the singly charged HSO_4_^−^ adducts. Fragmentation analysis of Cl-(TPP)2-CYSTAM and HSO_4_-(TPP)2-CYSTAM did not reveal structures analogous to the [M1c]^+^ ion. 

The identification of signals characterizing positively charged ions, present as TFA^−^, Cl^−^ and HSO_4_^−^ adducts, is difficult to explain as it would require additional charge formation or chemical bonds reorganization. Additionally, the *m*/*z* values of the signals presented on the obtained ESI-MS/MS spectra (Figure S1) in the case of the fragment ion corresponding to the TFA^−^, Cl^−^ and HSO_4_^−^ adducts are shifted by 1 Da in comparison to the noncovalent form of these adducts, which may suggest bond reorganization. The schematic presentation of the formed ions and corresponding *m*/*z* values are presented in Table 1. 

**Figure 4:**
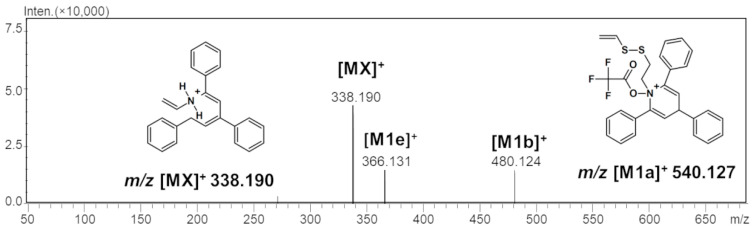
ESI-MS/MS/MS spectrum of TFA-(TPP)2-CYSTAM adduct. Parent ion 540.120 *m*/*z*; collision energy 50% *m*/*z*; range from 50 to 700.

Furthermore, ESI-MS/MS/MS analysis of an ion at *m*/*z* 540.127 revealed the formation of a fragment ion at *m*/*z* 480.116, which also corresponds to the TFA adduct (Figure 4). All these observations may suggest that the structure of the formed TFA-(TPP)2-CYSTAM and its fragments are stable in the gas phase. To test this hypothesis, computational analysis was performed.

### 2.2. Computational Analysis

**Figure 5:**
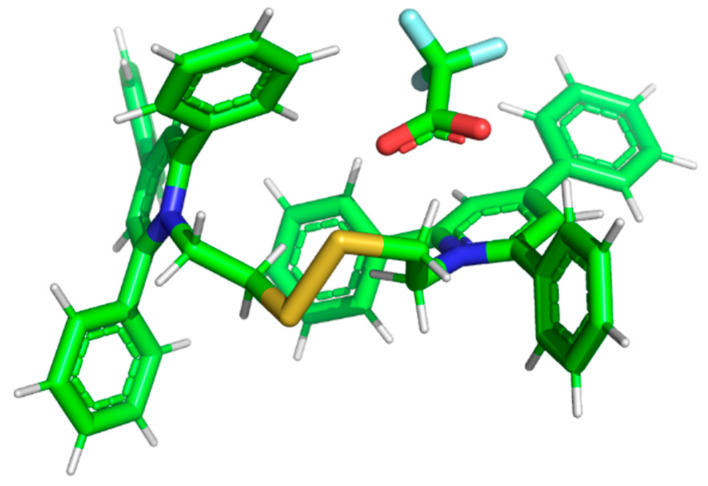
The structure of the TFA-(TPP)2-CYSTAM ion (M1^+^).

Computational methods of theoretical chemistry have been used as useful tools to predict the structure and properties of organic and inorganic compounds [38,39,40,41,42]. The molecular orbital studies on series TFA-(TPP)2-CYSTAM adduct were conducted on the DFT level of theory. The structure of the thermodynamically stable TFA-(TPP)2-CYSTAM (M1^+^ ion) adduct is presented in Figure 5. The total electronic energy (E_tot_) of the TFA-(TPP)2-CYSTAM equals −3362.437395 hartree. Please note that, in the TFA-(TPP)2-CYSTAM adduct (M1^+^ ion), we can distinguish a well-separated molecule from an ion; the O1..N distances are 3.109Å and O2..N 3.026Å. The full structure of the adduct is presented in the Supplementary Materials (Table S1).

**Figure 6:**
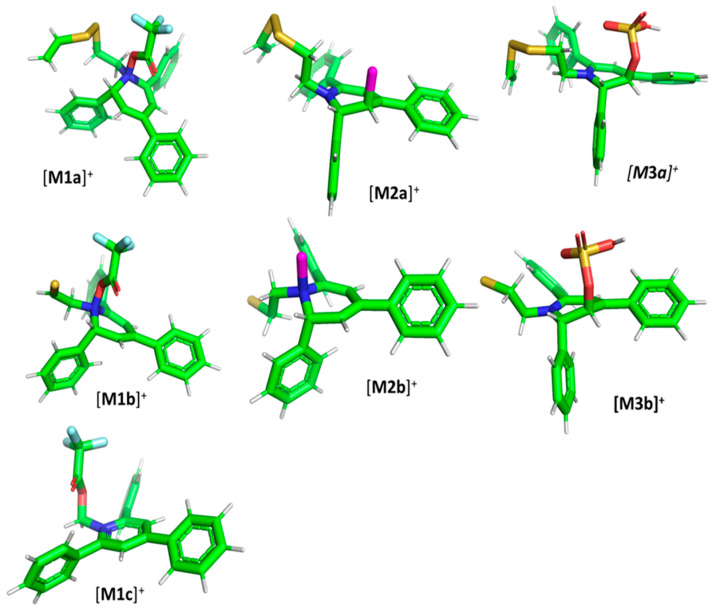
The structure of the [M1a]^+^, [M1b]^+^ and [M1c]^+^ ions (daughter ions of TFA-(TPP)2-CYSTAM); [M2a]^+^ and [M2b]^+^ (daughter ions of Cl-(TPP)2-CYSTAM) and [M3a]^+^ and [M3b]^+^ (daughter ions of HSO_4_^−^(TPP)2-CYSTAM).

The [M1a]^+^ adduct (Figure 6) with total electronic energy −2420.861483 hartree is formed via N–O single bond between an oxygen atom in trifluoroacetate and a nitrogen atom in TPP moiety with a length of 1.472 Å. Similar single bond lengths for N–O interactions can be found both in computational and experimental studies, e.g., on nitrosyl nitrite (1.471 Å) [43], dinitrogen pentoxide (1.498 Å) [44], nitrous acid (1.439 Å) [39] or 1,2-oxaziridine derivatives (1.495 Å) [45]. The complete Cartesia set of [M1a]^+^ data is presented in the Supplementary Materials (Table S2).

The formation of a N–O bond requires the reorganization of chemical bonds within the pyridinium moiety, which is associated with a loss of aromaticity. Additionally, the nitrogen atom is still positively charged, which makes the identification of the formed compound possible. 

Similarly to the [M1a]^+^ ion, the [M1b]^+^ (E_tot_ = −1944.024176 hartree) consists of a well-defined single bond between N and O atoms with a length of 1.475 Å. The fully optimized structure of the [M1b]^+^ ion is presented in Figure 6, and Cartesian coordinates can be found in the Supplementary Materials (Table S3). The [M1c]+ ion, with E_tot_ = −1506.601239 hartree, differs substantially from the longer [M1a]^+^ and [M1b]^+^ ions. Rather than via N–O, the TFA part is connected to the TPP through a carbon atom as presented in Figure 6. The C–O bond length (1.411 Å) is typical for a single bond between O and C. Please see the complete structure parameters of the [M1c]+ in the Supplementary Materials (Table S4).

The [M2a]^+^ ion displays total electronic energy equal to −2355.182614 hartree and bonds Cl^−^ anion using the C3 atom of the NC5 ring with a C–Cl distance of 1.858 Å as presented in Figure 6. In the [M2b]+, chlorine anion (E_tot_ = −1878.650926 hartree) bonds via nitrogen with N–Cl distance of 1.837 Å as presented in Figure 6. The cation bonds the HSO_4_^−^ anion regarding the C3 atom of the NC5 ring with a C–O distance of 1.495 Å as presented in Figure 6. The complete set of the Cartesian structure parameters of the [M3a]^+^ ion can be found in the SM. Similarly to [M3a]^+^ (E_tot_ = −2594.432952 hartree), the [M3b]^+^ (E_tot_ = −2118.378340 hartree) ion binds to the HSO_4_^−^ anion via the C3 atom of the NC5 ring (Figure 6). The C–O distance is slightly shorter (1.455 Å).

### 2.3. UV–Vis Absorption Analysis

**Figure 7:**
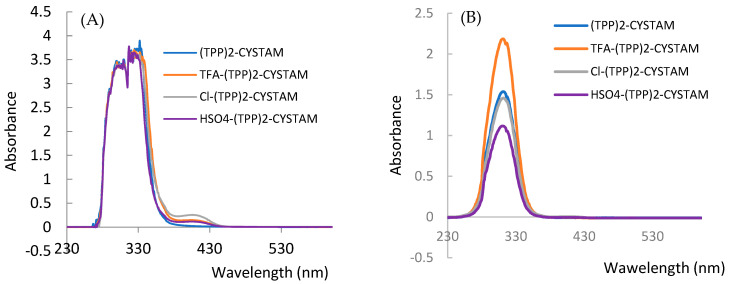
UV–Vis spectrum of (TPP)2-CYSTAM and its adducts with TFA, Cl^−^ and HSO_4_^−^.All samples were dissolved in MeOH. Panel (**A**): concentration of each sample was equal to 1.37 × 10^−3^. Panel (**B**): concentration of each sample was equal to 1.37 × 10^−4^ M.

To investigate the possible absorbance properties, we performed UV–Vis analysis and combined it with fluorescent analysis because application of both techniques helps to obtain more information about an analyzed compound. Figure 7A,B show the absorption spectra of bisquaternary ammonium dication and its adducts with TFA^−^, Cl^−^ and HSO_4_^−^ anions for TPP solutions of 10^−3^ mol/L and 10^−4^ mol/L, respectively. 

The absorption spectrum of TPP reveals a broad asymmetric band in the range 280–380 nm with a maximum at 310 nm (Figure 7) corresponding to the ^1^π*←^1^π transition of intramolecular charge transfer (ICT) nature from phenyl substituents on the heteroring [45]. The formation of adducts causes a change in intensity as well as the appearance of an additional weak band in the range 380–450 nm centered at 404 nm (Figure 7). The intensity of the additional band is highest for Cl-(TPP)2-CYSTAM. A pronounced hyperchromic effect is observed for the TFA-(TPP)2-CYSTAM adduct; the molar absorption coefficient increases from 1.40 × 10^4^ for (TPP)2-CYSTAM to 1.93 × 10^4^ for TFA-(TPP)2-CYSTAM. A hypochromic effect occurs for HSO_4_-(TPP)2-CYSTAM, with the extent of the change in the molar absorption coefficient being slightly smaller than for TFA-(TPP)2-CYSTAM. In contrast, a very weak hypochromic effect is observed for the adduct with the Cl^−^ ion. Changes in the intensity of the absorption bands for adducts as well as the appearance of bands in the lower energy range are the result of ion–TPP interactions. The strength and types of interactions as well as the nature of the anions are responsible for the magnitude of the observed changes. 

### 2.4. Analysis of Anion Sensor Properties

#### 2.4.1. Analysis of Luminescent Properties

**Figure 8:**
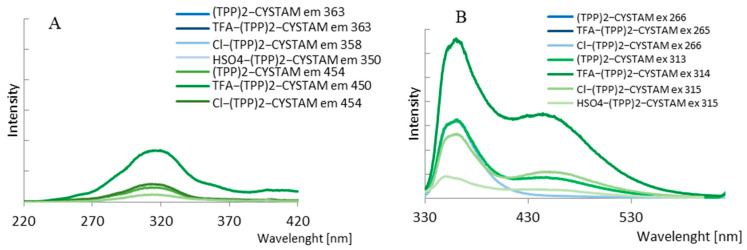
(**A**) Excitation and (**B**) emission spectra of (TPP)2-CYSTAM and its adducts with TFA^−^, Cl^−^ and HSO_4_^−^. Concentration of (TPP)2-CYSTAM = 1.37 × 10^−5^ M. Excitation was monitored at the emission wavelengths of 363 and 454 nm for (TPP)2-CYSTAM, 363 and 450 nm for T TFA-(TPP)2-CYSTAM, 358 and 454 nm for Cl-(TPP)2-CYSTAM and 350 and 450 nm for HSO_4_-(TPP)2-CYSTAM. The wavelengths of the excitation radiation λ = 266 and 313 nm for (TPP)2CYSATM, λ = 265 and 314 nm for TFA-(TPP)2-CYSTAM, λ = 266 and 315 nm for Cl-(TPP)2-CYSTAM and λ = 315 nm for HSO_4_-(TPP)2-CYSTAM.

The fluorescent properties of bisquaternary ammonium compound and its adducts with TFA^−^, Cl^−^ and HSO_4_^−^ were studied. Two different concentrations of (TPP)2-CYSTAM—1.37 × 10^−4^ M and 1.37 × 10^−5^ M were examined (Figure 8). The amount of added acids was, respectively, 1.31 × 10^−2^, 1.31 × 10^−3^ M in the case of TFA adduct, 3.24 × 10^−5^, 3.24 × 10^−6^ mole in the case of Cl-adduct and 1.87 × 10^−5^ and 1.87 × 10^−6^ mole in the case of HSO_4_-adduct. 

**Figure 9:**
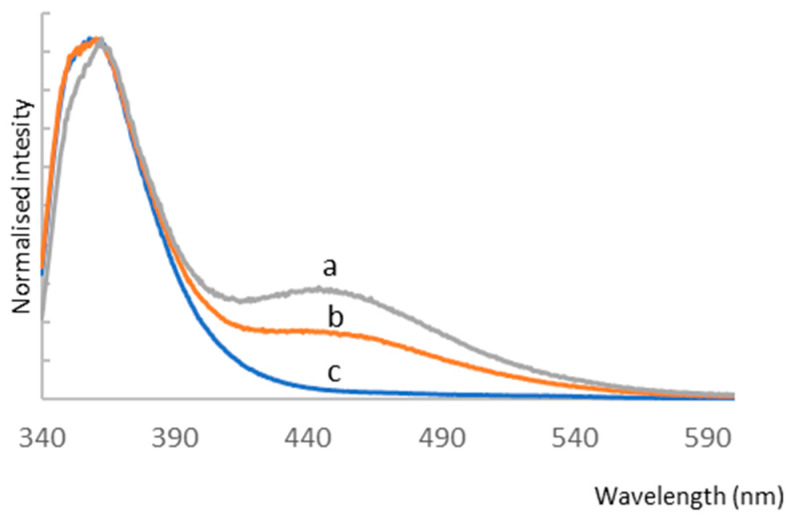
Emission spectra of (TPP)2-CYSTAM. Concentration: 1.37 × 10^−5^ M. λ_exc_ for a = 313, b = 330 and c = 266 nm.

Due to the presence of an internal filter effect for a 10^−4^ mol/L solution, only the results for a 10^−5^ mol/L sample were analyzed in this paper. The emission spectra of TPP are shown in Figure 9, and their spectral profiles depend on the excitation wavelength.

**Figure 10:**
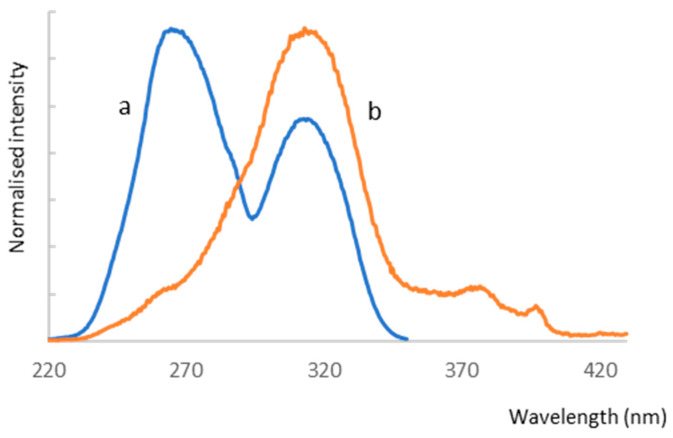
Excitation spectra of (TPP)2-CYSTAM. Concentration 1.37 × 10^−5^ M. λ_mon_ for a = 363 nm and b = 450 nm.

For λ_exc_ = 266 nm, the emission spectrum consists of one broad band (band I) with a maximum at 361.0 nm. For excitation with 313.5 and 330.0 nm, a second band (band II) appears in the spectral range of 415–550 nm with a maximum at 446.0 nm. The relative intensity ratio of band I to band II depends on λ_exc_ and decreases as the excitation wavelength shifts toward lower energy. Figure 10 shows the different profiles of the excitation spectra, which vary with emission wavelength. The excitation spectrum is dominated by a broad band with a maximum at 313.5 nm for λ_mon_ = 450 nm. In the spectral range of 360–410 nm, low-intensity bands are visible. Moreover, a second intense band appears in the excitation spectrum at λ_mon_ = 363.0 nm centered at 265 nm. Considering the excitation band located at lower energy in the spectrum recorded for λ_mon_ = 363 nm, the Stokes shift was calculated as 4210 cm^−1^ (Figure S2). This is much smaller than the anomalous Stokes shifts observed for 2,4,6-(N-alkyl)triarylsubstituted pyridinium cations, which were about 10,000 cm^−1^ and were caused by adiabatic structural relaxation (ASR) with the formation of the flattened structure [46,47,48]. Jacobian transformation was used to convert the excitation spectra (Figure S2) from wavelength to wavenumber. The use of Jacobian transformation is important because the shape and position of spectral features can vary significantly depending on the unit of measure used [49]. The nature of the changes in emission and excitation spectra testifies to the complex process of depopulation of (TPP)2-CYSTAM excited states.

**Figure 11:**
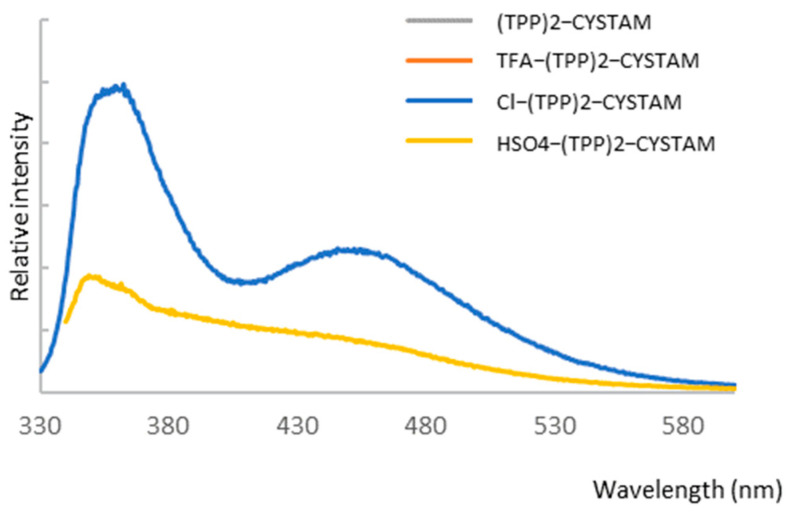
Emission spectra of (TPP)2-CYSTAM and its adducts with TFA^−^, Cl^−^ and HSO_4_^−^. Concentration of (TPP)2-CYSTAM = 1.37 × 10^−5^ M. For each sample, λ_exc_ = 313.5 nm.

The emission properties of (TPP)2-CYSTAM, as well as the varying effects of anions on its absorption properties, prompted us to study the luminescence detection capability of (TPP)2-CYSTAM toward the anions. Only one of the anions, TFA, caused an increase in (TPP)2-CYSTAM luminescence while significantly increasing the ratio of the relative intensity of the emission band at lower energy to that of the higher energy band (Figure 11). 

**Figure 12:**
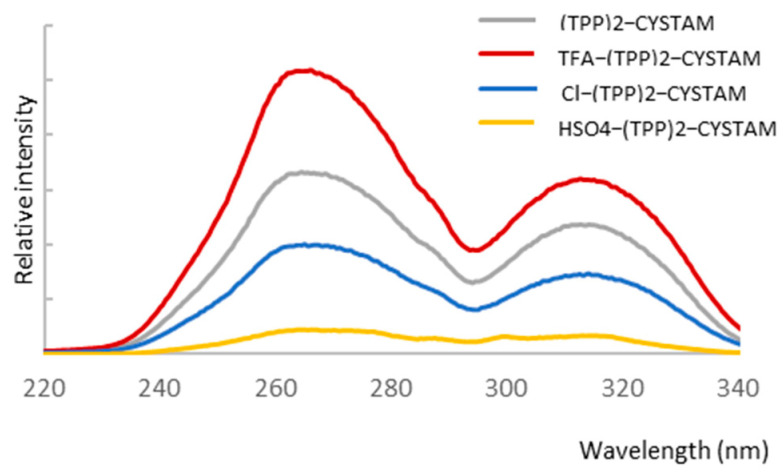
Excitation spectra of (TPP)2-CYSTAM and its adducts with TFA^−^, Cl^−^ and HSO_4_^−^. Concentration of (TPP)2-CYSTAM = 1.37 × 10^−5^ M. For each sample, λ_em_ = 360 nm.

A smaller effect of increasing the emission intensity is observed with excitation at λ = 266 nm as shown in Figure S3. Sensory properties in the direction of the anion to selectively increase the intensity of the analyte’s luminescence are highly desirable due to the fact that a large number of compounds contribute to a decrease in signal intensity. The other anions tested reduced the emission intensity to varying degrees. This is illustrated by the emission spectra in Figure 11. Analogous intensity changes are presented in the excitation spectra in Figure 12.

**Figure 13:**
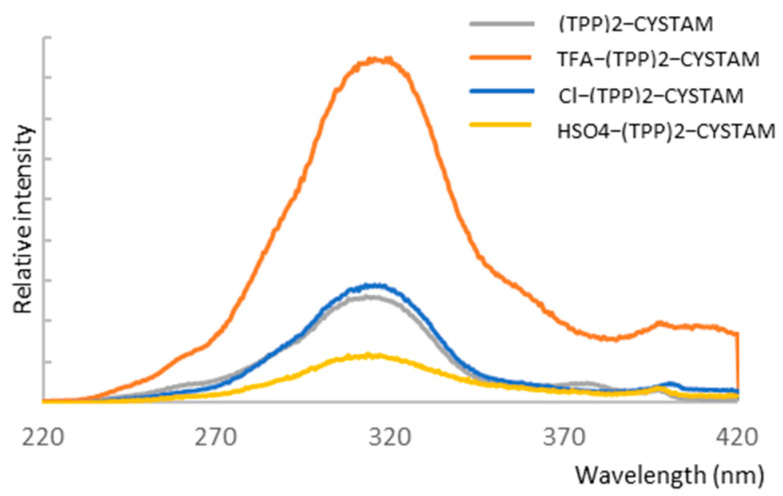
Excitation spectra of (TPP)2-CYSTAM and its adducts with TFA^−^, Cl^−^ and HSO_4_^−^. Concentration of (TPP)2-CYSTAM = 1.37 × 10^−5^ M. For each sample, λ_em_ = 450 nm.

A particularly large difference in the intensity of the excitation band of the TFA-(TPP)2-CYSTAM adduct compared to (TPP)2-CYSTAM is observed for λ_mon_ = 450 nm (Figure 13), which is consistent with the emission spectra in Figure 11. Of note is the very large increase in the emission centered at 450 nm generated by the interaction of (TPP)2-CYSTAM with the TFA ion.

#### 2.4.2. Sensitivity of Anion Sensing

Due to the growing importance of anion sensors, we decided to check the possibility of using bisquaternary ammonium dication as a sensor for trifluoroacetate anion. We prepared eight samples of (TPP)2-CYSTAM. Each sample was dissolved in 999 µL methanol. Before measurement, each sample was incubated with 1 µL of an appropriate solution of trifluoroacetic acid (Table 2) for 10 min.

**Figure 14:**
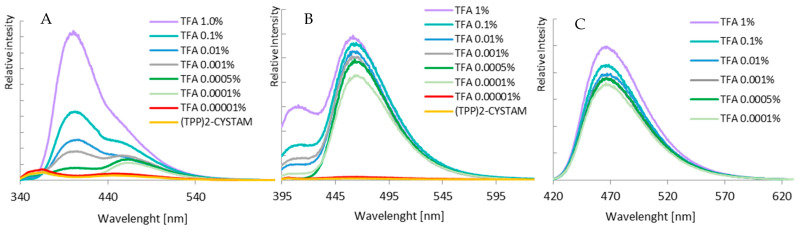
Emission spectra of a (TPP)2-CYSTAM depending on the amount of TFA. Excitation wavelengths: (**A**) 330 nm, (**B**) 358 nm and (**C**) 408 nm.

The sample preparation procedure was modified due to the need to quantitatively compare changes in emission intensity after anion addition. A remarkable difference in (TPP)2-CYSTAM luminescence response in terms of intensity and profile was detected in the presence of TFA. As demonstrated in Figure 14A, the addition of a minimal amount of TFA (0.00001% concentration) causes an increase in emission intensity with no change in the luminescence profile, while the addition of 0.0001% TFA concentration causes a dramatic increase in intensity with a change in the emission profile (one band centered at 466 nm dominates). The addition of 0.001% TFA, in addition to an increase in luminescence, causes a change in profile, which is slightly different than for the 0.0001% concentration as a second band appears with a maximum at 402 nm. A further increase in intensity and change in profile appear at concentrations of 0.01, 0.1 and 1%. The amount of 1% TFA causes a sharp increase in luminescence intensity. Thus, the presence of TFA not only enhances the intensity but is responsible for tuning the color of the luminescence. The excitation wavelength was chosen so that it covers the excitation band of the free (TPP)2-CYSTAM solution as well as the TFA-(TPP)2-CYSTAM adduct and provides the (TPP)2-CYSTAM luminescence response with a drastically small amount of TFA. 

An analogous detection range can be obtained by exciting samples with a wavelength of 358 nm. This wavelength lies at the end of the range of the free (TPP)2-CYSTAM excitation band and at the slope of the TFA-(TPP)2-CYSTAM adduct excitation band. In this case, there is an increase in signal intensity for 0.00001% TFA, and then a sharp increase in emission intensity with a change in profile for 0.0001% TFA, as demonstrated in Figure 14B.

In addition, in studying the luminescence response of (TPP)2-CYSTAM toward the TFA anion, we also used excitation with a wavelength of 408 nm, which does not cover the range of the excitation band for free (TPP)2-CYSTAM. This is the maximum of the excitation band of the TFA-(TPP)2-CYSTAM adduct. Such measurement conditions allow detection of TFA at 0.0001% concentration, for which a clear luminescence band appears, as shown in Figure 14C. Addition of TFA from 0.001% to 1% results in a further increase in luminescence signal. 

**Figure 15:**
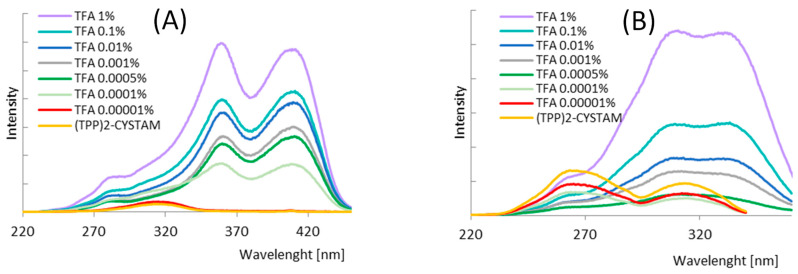
Excitation spectra of a (TPP)2-CYSTAM depending on the amount of TFA. Excitation spectra monitored at an emission wavelength of (**A**) 466 nm; (**B**) for TFA amount 1–0.0005%—403 nm, for 0.0001% TFA, 0.00001% TFA and (TPP)2-CYSTAM—366 nm.

The effect of the TFA anion indicates a very sensitive “turn-on” luminescence response of (TPP)2-CYSTAM for TFA in solution. Moreover, detection can also be carried out based on the increase in intensity and change in profile of excitation spectra by monitoring emissions at different wavelengths. A very low concentration of TFA results in a drastic change in the excitation spectrum profile, with an increase in intensity and range toward lower energies up to 455 nm, and also the appearance of new bands with maxima of 282, 359 and 410 nm (for λ_mon_ = 466 nm; Figure 15A). Figure 15B presents the changes in intensity and profile of excitation spectra for λ_mon_ = 403 nm and 366 nm.

The observed differences in the emission and excitation spectra testify to the sensitivity of bisquaternary ammonium dication to trifluoroacetate anion even in the amount of 1.3 × 10^−8^ mole (1.3 × 10^−5^ M), which is equal to 1.469 ppm. 

Several TFA sensors have been studied. The tetraphenylethylene (TPE)-planarized bis-Schiff-base as a “turn-on”-type fluorescence sensor was evaluated during the test paper of the sensor being hung in the TFA vapor or in the low-concentration solution of TFA (1 × 10^−5^ M). This sensor exhibited obvious fluorescent emission changes [50]. In different studies, the limit of detection for TFA with use of a probe in the form *ortho*-hydroxyphenyl to pyrazoline with a benzothiazole backbone determined by HPLC with a UV detector was equal to 1.3 µg L^−1^ (0.00130 ppm) [51]. Consequently, we believe that our findings are highly promising and open the way to further research regarding sensitivity to the TFA anion.

It can be inferred that the proposed anion receptors possess significant advantages over those previously presented in the literature due to their simplicity of synthesis, ability to bind anions and ability to be analyzed using two distinct analytical techniques. This approach provides a deeper understanding of the chemical nature of cation–anion interactions as well as a more accurate characterization.

## 3. Materials and Methods

### 3.1. Chemicals

All chemicals were used as supplied. Tetrafluoroborate 2,4,6-triphenylpyrylium (TPP), cystamine dihydrochloride, triethylamine (TEA), dimethylformamid (DMF), acetonitrile (MeCN), water (LC/MS grade), formic acid (HCOOH), trifluoroacetic acid (TFA), hydrochloric acid (HCl) and sulfuric acid (H_2_SO_4_) were purchased from Sigma-Aldrich (St. Louis, MO, USA).

### 3.2. (TPP)2-CYSTAM Synthesis

Synthesis of (TPP)2-CYSTAM was performed by dissolving 22.5 mg (0.1 mmol) of cystamine dichydrochloride and 39.6 mg (0.1 mmol) TPP salt in 1 mL of the mixture of DMF/water (4:1). Then, 56.1 μL of *N*,*N*,*N*-trithylamine (TEA) (0.4 mmol, 4-fold excess) was introduced. The reaction was carried out at room temperature for two hours. Afterward, the product was lyophilized. A small amount of the (TPP)2-CYSTAM was re-dissolved in the mixture of H_2_O:MeCN:HCOOH (50:50:0.1) and analyzed by ESI-MS. The remaining amount of compound was dissolved in the mixture of H_2_O:MeCN (60:40 *v*/*v*) and purified by HPLC.

### 3.3. TFA, Cl and HSO_4_ Bisquaternary Ammonium Dication Adduct Preparation

Synthesis of TFA-(TPP)2-CYSTAM, Cl-(TPP)2-CYSTAM and HSO_4_-(TPP)2-CYSTAM was performed by dissolving 5 mg of (TPP)2-CYSTAM in 999 μL of MeCN for each sample. Moreover, 1 μL of the appropriate acid solution was added to the samples. The samples were incubated for an hour. Afterward, the product was lyophilized.

### 3.4. Mass Spectrometry

ESI-MS/MS and ESI-MS/MS/MS experiments for (TPP)2-CYSTAM and its adduct with TFA were performed on the Shimadzu LCMS-IT-TOF (Shimadzu, Kyoto, Japan) system equipped with Nexera X2 chromatographic module. Analyses were performed in the positive ion mode between 50 and 1000 *m*/*z*. ESI-MS parameters: nebulizing gas—nitrogen, nebulizing gas flow—3.0 L/min, drying gas flow—10 L/min, heating gas flow—10 L/min, interface temperature 300 °C, desolvation line temperature—400 °C, detector voltage—2.02 kV, interface voltage—4.0 kV, collision gas—argon and collision energy was optimized between 30 and 60% for IT-TOF. The injection volume was optimized depending on the intensity of the signals observed on the mass spectrum within the range of 0.1 to 1 μL. All obtained signals had a mass accuracy error in the range of 1 ppm. All the used solvents were of LC–MS grade. The LC module was equipped with water +0.1% HCOOH as a mobile phase, eluent B: acetonitrile +0.1% HCOOH. Flow rate—0.3 mL/min. The obtained data were analyzed by LabSolutions 4.0 software (Shimadzu, Kyoto, Japan).

ESI-MS and ESI-MS/MS experiments for Cl-(TPP)2-CYSTAM and HSO_4_-(TPP)2-CYSTAM were performed on a Bruker Compact mass spectrometer (Bruker Daltonics, Bremen, Germany) equipped with standard ESI source. The instrument was operated in the positive ion mode and calibrated with the Tunemix™ mixture (Agilent Technologies, Palo Alto, CA, USA). The mass accuracy was better than 5 ppm. Analyte solution was infused at a flow rate of 3 μL/min. The instrument parameters were as follows: scan range: 200–3000 *m*/*z*; drying gas: nitrogen; flow rate: 3.0 L/min; temperature: 200 °C; nebulizing gas: nitrogen; 0.3 Bar; potential between the spray needle and the orifice: 4.0 kV. For MS spectra analysis, Bruker Compass DataAnalysis 4.0 software was used.

### 3.5. High-Performance Liquid Chromatography

All crude products were purified by HPLC using a Varian Microsorb-MV 100^−5^ CN column (4.6 mm × 250 mm; Varian, Palo Alto, CA, USA) with a gradient elution of 50–100% B in A (A = 0.1% TFA in water; B = 0.1% TFA in acetonitrile) for 40 min (flow rate 7 mL/min).

### 3.6. Computational Analysis

The molecular orbital studies on series TFA-(TPP)2-CYSTAM adduct have been completed on the DFT level of theory. Gaussian 16 C.01 [52] suite of programs using the ωB97X-D [53] long-range corrected hybrid density functional with damped atom–atom dispersion corrections was used with triple-ζ 6-311G(2d,2p) basis set. The presented structure was fully optimized with demanding convergence criteria (RMS Force = 1 × 10^−5^, RMS Displacement = 4 × 10^−5^, Max Force = 2 × 10^−5^ and Max Displacement = 6 × 10^−5^) predefined as “opt = tight” in the Gaussian package in atomic units. The graphic work was completed with PyMOL [54] program. Please note that all presented structures are thermodynamically stable.

### 3.7. UV–Vis Measurement

For UV–Vis measurement, each sample was dissolved in MeOH—1 mg of (TPP)2-CYSTAM, 1.1 mg of TFA-(TPP)2-CYSTAM, 1 mg of Cl-(TPP)2-CYSTAM and 1.1 mg of HSO_4_-(TPP)2-CYSTAM; a concentration 1.37 × 10^−3^ M has been achieved. For concertation 1.37 × 10^−4^, all samples were 10-fold diluted. For measurement, 200 µL of each solution was used. Absorption spectra were recorded on a Tecan Nanoquant Infinite M200PRO spectrophotometer. Absorbance scan parameters: wavelength from 230 to 500 nm, step 1 nm, number of flashes 15 and settle time 0. 

### 3.8. Emission and Excitation Measurement

For emission and excitation measurement, each sample was dissolved in 1000 µL of MeOH—1 mg of (TPP)2-CYSTAM, 1.1 mg of TFA-(TPP)2-CYSTAM, 1 mg of Cl-(TPP)2-CYSTAM and 1.1 mg of HSO_4_-(TPP)2-CYSTAM; a concentration of 1.37 × 10^−3^ M has been achieved. For samples with lower concentrations, appropriate dilutions were created—10-fold for 1.37 × 10^−4^ M and 100-fold for 1.37 × 10^−5^ M. We prepared three different concentrations to check whether the inner filter effect appears in the case of the higher concentrations of the analyzed compound. For analysis of sensitivity of an anion sensor, 1 mg of (TPP)2-CYSTAM was dissolved in 1000 μL of MeOH, and 989 µL MeOH was prepared in 8 Eppendorf tubes, 10 µL of (TPP)2-CYSTAM was introduced into each of them and 1 μL of TFA aqueous solution (in a proper concentration) was added 10 min before measurement. Emission and excitation measurements were performed using an Edinburgh Instruments FLSP 920 spectrofluorometer equipped with Hamamatsu R-928 photomultiplier and a 450 W Xe lamp as an excitation source. These spectra were measured at 295 K in quartz cuvettes. The emission and excitation spectra were corrected for the instrument response. All measurements were conducted using appropriate optical filters.

## 4. Conclusions

In conclusion, we demonstrated the possibility of identification of stable noncovalent TFA-bisquaternary ammonium dication, Cl-bisquaternary ammonium dication and HSO_4_^−^ bisquaternary ammonium dication during ESI-MS analysis. The MS/MS analysis revealed that the noncovalent adducts undergo transformation into a covalent system in the presence of collision energy. The TFA anion forms a N–O bond with the analyzed molecule, the Cl^−^ anion forms a Cl–C bond with the C3 atom of the NC5 ring or N–Cl bond depending on the daughter ion and the HSO_4_^−^ anion forms a C–O bond with the C3 atom of the NC5 ring. Such phenomena result in bond reorganization and charge retention on the nitrogen atom, which allows its observation in positive ESI-MS mode. It is demonstrated that it is possible to observe the TFA^−^, Cl^−^ and HSO_4_^−^ adducts in the ESI-MS analysis, while also showing the possibility of formation of new chemical bonds in the gas phase induced by collision. This significantly broadens the knowledge of mass spectrometry and intermolecular detachments. We have also proved that the influence of the TFA anion indicates a very sensitive luminescence response of the derived dication for TFA in solution, with detection as high as 1.3 × 10^−5^ M. We determined that detection can be carried out by varying the intensity and profile of both the emission and excitation spectra, and the emission color can be tuned depending on the wavelength of the excitation radiation. The rapid response, high sensitivity and adequate stability of the bisquaternary ammonium compound make it a promising candidate for use as a “turn-on” luminescence sensor for TFA anion recognition. The conducted research significantly enhances the potential of mass spectrometry in identifying interactions between cations and anions in the gas phase, thereby enabling the utilization of this technique in identifying specific anions in samples.

## Figures and Tables

**Table 1:** Structure of identified ions formed during the ESI-MS/MS experiment. Collision energy 50%.

TFA-(TPP)2-CYSTAM Parent Ion: *m*/*z* 847.270 (M1^+^)	Cl-(TPP)2-CYSTAM Parent Ion: *m*/*z* 769.245 (M2^+^)	HSO_4_-(TPP)2-CYSTAM Parent Ion: *m*/*z* 831.238 (M3^+^)
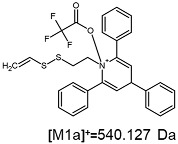	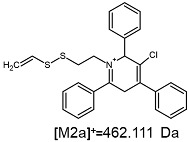	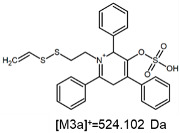
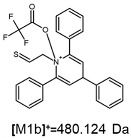	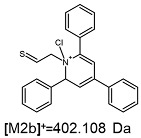	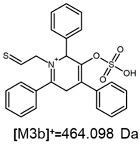
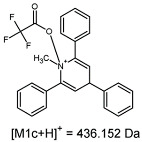	-	-
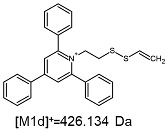	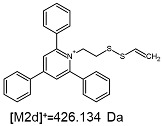	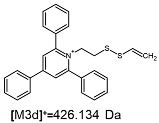
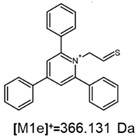	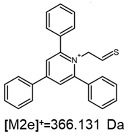	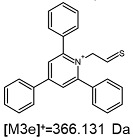
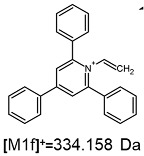	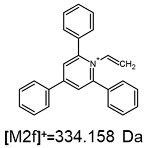	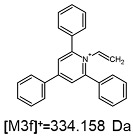
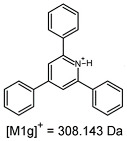	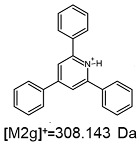	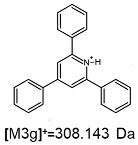
-	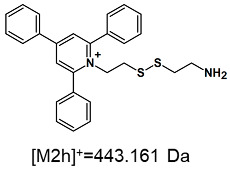	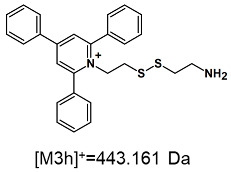

**Table 2:** Amount of added TFA in analyzed samples.

	Amount of TFA in the Analyzed Sample	
Sample	Percent Concentrations [%]	Amount of Mole
1	1	1.31 × 10^−4^
2	0.1	1.31 × 10^−5^
3	0.01	1.31 × 10^−6^
4	0.001	1.31 × 10^−7^
5	0.0005	0.655 × 10^−7^
6	0.0001	1.31 × 10^−8^
7	0.00001	1.31 × 10^−9^

## Data Availability

Data are contained within the article and Supplementary Materials.

## Supplementary Materials

The following supporting information can be downloaded at https://www.mdpi.com/article/10.3390/ijms25063467/s1.
