# Supplementary material for: A Novel Bisquaternary Ammonium Compound as an Anion Sensor—ESI-MS and Fluorescence Study
Source: Int J Mol Sci. 2024 Mar 19;25(6):3467. doi: 10.3390/ijms25063467 (PMC10970393; doi:10.3390/ijms25063467)
Supplement: Supplementary file 1 [file ijms-25-03467-s001.zip › ijms-2888654-supplementary.pdf]

A Novel Bisquaternary Ammonium Compound as an Anion Sensor - ESI-MS and Fluorescence Study

Marta Kowalska, Robert Wieczorek, Paula Gawryszewska and Remigiusz Bąchor*

Faculty of Chemistry, University of Wroclaw, F. Joliot-Curie 14, 50-383 Wroclaw, Poland.
*Correspondence: remigiusz.bachor@uwr.edu.pl; Tel.: +48-71-375-7212; Fax: +48-71-328-2348

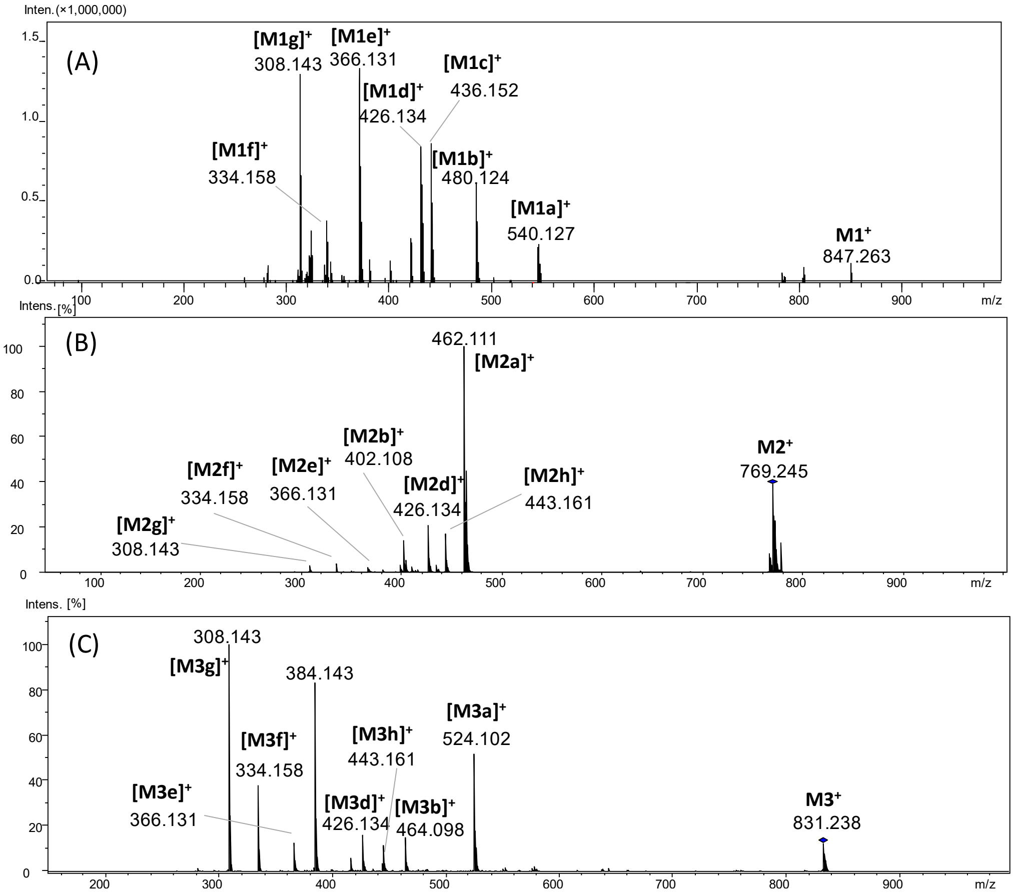

Figure S1. ESI-MS/MS spectrum, of A) TFA-(TPP)2-CYSTAM adduct, B) Cl-(TPP)2-CYSTAM, C) HSO_4-(TPP)2-CYSTAM adduct in the positive mode. Parent ion *m/z* A) 847.270, B) 769.245, C) 831.238 collision energy 50 %, *m/z* range from A) 50 to 1000, B) 50-1000 and C) 150-100.

Table S1. Cartesian set of [**M1**]$^+$

C	-2.117114	-0.265483	-1.415909
C	-2.932816	-1.36984	-1.494613
C	-4.304417	-1.27759	-1.272792

C	-4.813299	-0.01007	-1.026169
H	-2.483063	-2.313498	-1.760552
H	-5.86745	0.141907	-0.854352
C	-0.679526	-0.421163	-1.7609
C	-0.133998	0.256339	-2.848983
C	0.095047	-1.344004	-1.063111
C	1.173503	0.012409	-3.237854
H	-0.73726	0.961984	-3.405274
C	1.401056	-1.586984	-1.458176
H	-0.330444	-1.841852	-0.204617
C	1.939777	-0.91423	-2.545074
H	1.581943	0.52954	-4.095509
H	1.990678	-2.321541	-0.926975
H	2.950545	-1.13011	-2.865741
C	-5.172838	-2.465092	-1.32301
C	-4.730316	-3.67726	-0.794423
C	-6.445874	-2.38798	-1.887185
C	-5.551758	-4.790831	-0.828398
H	-3.763234	-3.736935	-0.314929
C	-7.257874	-3.507822	-1.932245
H	-6.7928	-1.457483	-2.317051
C	-6.812902	-4.709858	-1.400738
H	-5.209597	-5.722014	-0.399007
H	-8.238182	-3.443104	-2.38328
H	-7.451028	-5.582241	-1.429331
C	-1.719373	2.077549	-0.794337
H	-1.803129	2.803677	-1.598607
H	-0.731519	1.650919	-0.839833
C	-1.91716	2.739358	0.566854
H	-2.534127	3.627529	0.491727
H	-2.365515	2.030523	1.265309
S	-0.354431	3.229952	1.392623
S	0.937472	3.718949	-0.137074
N	-2.643336	0.946654	-1.087751
O	-1.264314	-0.246377	1.326569
C	-2.412484	-0.525984	1.719987
O	-3.411471	0.188521	1.827656
C	-2.585941	-2.017538	2.135578
F	-1.983993	-2.261749	3.310438
F	-2.006941	-2.851208	1.231343
F	-3.854817	-2.397179	2.240465
C	4.581981	0.58575	-0.40207
C	3.399943	-0.110792	1.526214

C	4.985958	-0.707802	-0.629356
C	3.793418	-1.411676	1.2991
C	4.56676	-1.753218	0.194655
H	5.662235	-0.888041	-1.451241
H	3.457025	-2.161075	1.999498
C	2.607871	0.230668	2.729712
C	3.214572	0.93524	3.770964
C	1.285646	-0.175354	2.831384
C	2.487726	1.23253	4.90894
H	4.250048	1.241841	3.690463
C	0.5608	0.137792	3.972503
H	0.783183	-0.68595	2.021596
C	1.158984	0.834888	5.008194
H	2.956411	1.772459	5.719773
H	-0.474201	-0.16466	4.030771
H	0.591721	1.074578	5.896836
C	4.928922	-3.150584	-0.091905
C	5.172377	-4.052247	0.944826
C	5.012276	-3.593765	-1.412807
C	5.499864	-5.366946	0.663617
H	5.137224	-3.720308	1.973785
C	5.324868	-4.913018	-1.690338
H	4.804928	-2.91308	-2.228193
C	5.572628	-5.800095	-0.652738
H	5.70156	-6.054391	1.472879
H	5.372888	-5.25008	-2.716185
H	5.822929	-6.829106	-0.869823
C	5.070381	1.682335	-1.274733
C	4.715173	1.715485	-2.618688
C	5.941421	2.643128	-0.765725
C	5.209174	2.715239	-3.442717
H	4.041625	0.967402	-3.013614
C	6.437422	3.635693	-1.593465
H	6.239304	2.606705	0.274422
C	6.067812	3.675639	-2.930972
H	4.924269	2.741884	-4.485114
H	7.117343	4.37565	-1.19555
H	6.454547	4.453228	-3.574545
N	3.757656	0.863971	0.645947
C	3.061835	2.177857	0.69778
H	2.688211	2.316375	1.704171
H	3.772913	2.965602	0.480631
C	1.908904	2.183277	-0.297157

H	2.273297	2.146216	-1.323318
H	1.258839	1.325905	-0.126456
C	-3.984816	1.083532	-0.923469
C	-4.569161	2.408646	-0.620279
C	-5.207583	2.597305	0.60155
C	-4.516128	3.439676	-1.553009
C	-5.768961	3.829743	0.8943
H	-5.209597	1.791576	1.323601
C	-5.082876	4.667735	-1.254131
H	-4.044473	3.280524	-2.514445
C	-5.703685	4.864308	-0.028186
H	-6.252371	3.983799	1.84882
H	-5.045962	5.468714	-1.97923
H	-6.141765	5.824844	0.205338

Table S2. Cartesian set of [**M1a**]$^{+}$

C	0.920000	-0.951000	0.944000
C	2.103000	-0.512000	0.508000
C	2.318000	0.596000	-0.407000
C	1.258000	1.341000	-0.714000
H	2.969000	-1.051000	0.870000
H	1.316000	2.168000	-1.407000
C	0.765000	-2.092000	1.877000
C	1.244000	-1.960000	3.179000
C	0.195000	-3.300000	1.476000
C	1.152000	-3.019000	4.069000
H	1.690000	-1.025000	3.493000
C	0.100000	-4.353000	2.370000
H	-0.169000	-3.427000	0.466000
C	0.576000	-4.214000	3.666000
H	1.530000	-2.909000	5.075000
H	-0.338000	-5.288000	2.050000
H	0.503000	-5.040000	4.360000
C	3.664000	0.820000	-0.970000
C	4.443000	-0.260000	-1.381000
C	4.164000	2.113000	-1.110000
C	5.696000	-0.049000	-1.932000
H	4.058000	-1.269000	-1.299000
C	5.419000	2.322000	-1.658000
H	3.579000	2.956000	-0.765000
C	6.186000	1.242000	-2.071000
H	6.288000	-0.892000	-2.258000

H	5.803000	3.328000	-1.755000
H	7.166000	1.405000	-2.497000
C	-0.154000	2.235000	1.055000
C	0.693000	2.242000	2.161000
C	-1.039000	3.292000	0.868000
C	0.617000	3.267000	3.088000
H	1.422000	1.456000	2.297000
C	-1.097000	4.329000	1.787000
H	-1.693000	3.306000	0.005000
C	-0.278000	4.313000	2.903000
H	1.270000	3.259000	3.949000
H	-1.788000	5.145000	1.628000
H	-0.327000	5.116000	3.625000
C	-1.387000	-0.315000	1.553000
H	-0.907000	0.100000	2.436000
H	-1.550000	-1.374000	1.713000
C	-2.710000	0.400000	1.321000
H	-3.243000	0.263000	2.265000
H	-2.599000	1.470000	1.196000
S	-3.876000	-0.223000	0.068000
S	-3.441000	0.900000	-1.610000
C	-4.266000	2.431000	-1.269000
C	-3.806000	3.587000	-1.718000
H	-4.382000	4.492000	-1.585000
H	-2.873000	3.667000	-2.263000
H	-5.206000	2.338000	-0.743000
N	-0.308000	-0.257000	0.475000
O	-0.966000	-1.061000	-0.568000
C	-0.320000	-1.471000	-1.694000
O	0.740000	-1.159000	-2.100000
C	-1.263000	-2.488000	-2.400000
F	-0.634000	-2.998000	-3.434000
F	-2.372000	-1.886000	-2.808000
F	-1.590000	-3.467000	-1.557000
C	-0.062000	1.178000	-0.038000
H	-0.887000	1.323000	-0.734000

Table S3. Cartesian set of [**M1b**]$^{+}$

C	-0.125000	0.177000	1.215000
C	1.206000	0.220000	1.211000
C	2.043000	0.288000	0.019000
C	1.434000	0.580000	-1.131000
H	1.688000	0.218000	2.179000
H	1.972000	0.657000	-2.064000
C	-0.962000	0.104000	2.433000
C	-1.224000	1.267000	3.153000
C	-1.466000	-1.118000	2.880000
C	-1.990000	1.211000	4.307000
H	-0.827000	2.212000	2.807000
C	-2.234000	-1.167000	4.032000
H	-1.250000	-2.024000	2.330000
C	-2.497000	-0.004000	4.743000
H	-2.187000	2.114000	4.866000
H	-2.619000	-2.115000	4.380000
H	-3.094000	-0.047000	5.644000
C	3.497000	0.059000	0.145000
C	3.981000	-0.918000	1.013000
C	4.401000	0.806000	-0.608000
C	5.342000	-1.153000	1.116000
H	3.293000	-1.517000	1.597000
C	5.761000	0.572000	-0.502000
H	4.041000	1.590000	-1.262000
C	6.234000	-0.409000	0.358000
H	5.706000	-1.920000	1.785000
H	6.455000	1.161000	-1.085000
H	7.297000	-0.589000	0.442000
C	-0.341000	2.349000	-1.152000
C	0.201000	3.183000	-0.175000
C	-1.155000	2.897000	-2.139000
C	-0.107000	4.533000	-0.164000
H	0.876000	2.788000	0.569000
C	-1.451000	4.252000	-2.135000
H	-1.553000	2.268000	-2.924000
C	-0.937000	5.069000	-1.140000
H	0.317000	5.172000	0.597000
H	-2.076000	4.667000	-2.912000
H	-1.168000	6.125000	-1.134000
C	-2.237000	0.544000	-0.015000
H	-2.212000	1.591000	0.281000
H	-2.674000	-0.014000	0.813000

S	-2.936000	-0.397000	-2.592000
N	-0.806000	0.095000	-0.110000
O	-0.958000	-1.358000	-0.313000
C	-0.370000	-2.085000	-1.305000
O	0.388000	-1.760000	-2.144000
C	-0.890000	-3.537000	-1.097000
F	-0.388000	-4.310000	-2.030000
F	-2.216000	-3.567000	-1.157000
F	-0.505000	-3.976000	0.100000
C	-0.026000	0.864000	-1.216000
H	-0.432000	0.464000	-2.137000
C	-3.137000	0.367000	-1.197000
H	-4.099000	0.832000	-0.980000

Table S4. Cartesian set of [**M1c**]$^+$

C	-0.407000	-0.009000	0.949000
C	0.853000	-0.421000	1.098000
C	1.880000	-0.407000	0.066000
C	1.614000	0.247000	-1.064000
H	1.115000	-0.816000	2.071000
H	2.320000	0.286000	-1.882000
C	-1.433000	-0.026000	2.011000
C	-1.125000	0.559000	3.239000
C	-2.674000	-0.640000	1.838000
C	-2.039000	0.522000	4.280000
H	-0.171000	1.051000	3.373000
C	-3.585000	-0.669000	2.879000
H	-2.934000	-1.104000	0.897000
C	-3.270000	-0.090000	4.100000
H	-1.791000	0.978000	5.228000
H	-4.541000	-1.153000	2.739000
H	-3.984000	-0.117000	4.911000
C	3.148000	-1.125000	0.308000
C	3.139000	-2.371000	0.934000
C	4.362000	-0.574000	-0.096000
C	4.324000	-3.057000	1.140000
H	2.202000	-2.822000	1.233000
C	5.546000	-1.261000	0.115000
H	4.382000	0.406000	-0.556000
C	5.529000	-2.503000	0.732000

H	4.307000	-4.028000	1.615000
H	6.484000	-0.822000	-0.195000
H	6.454000	-3.038000	0.898000
C	0.638000	2.530000	-1.032000
C	1.375000	2.978000	0.061000
C	0.149000	3.449000	-1.955000
C	1.589000	4.334000	0.243000
H	1.789000	2.271000	0.767000
C	0.381000	4.804000	-1.782000
H	-0.418000	3.106000	-2.811000
C	1.094000	5.247000	-0.678000
H	2.156000	4.678000	1.096000
H	0.006000	5.511000	-2.508000
H	1.273000	6.304000	-0.541000
N	-0.807000	0.534000	-0.375000
O	-1.556000	-0.435000	-1.161000
C	-1.135000	-1.722000	-1.348000
O	-0.123000	-2.232000	-1.035000
C	-2.302000	-2.430000	-2.090000
F	-3.379000	-2.451000	-1.307000
F	-1.947000	-3.658000	-2.383000
F	-2.600000	-1.771000	-3.205000
C	0.379000	1.055000	-1.256000
H	-0.028000	0.921000	-2.256000
C	-1.789000	1.638000	-0.186000
C	-2.906000	1.760000	-0.863000
H	-3.531000	2.615000	-0.649000
H	-3.240000	1.055000	-1.606000
H	-1.443000	2.325000	0.567000

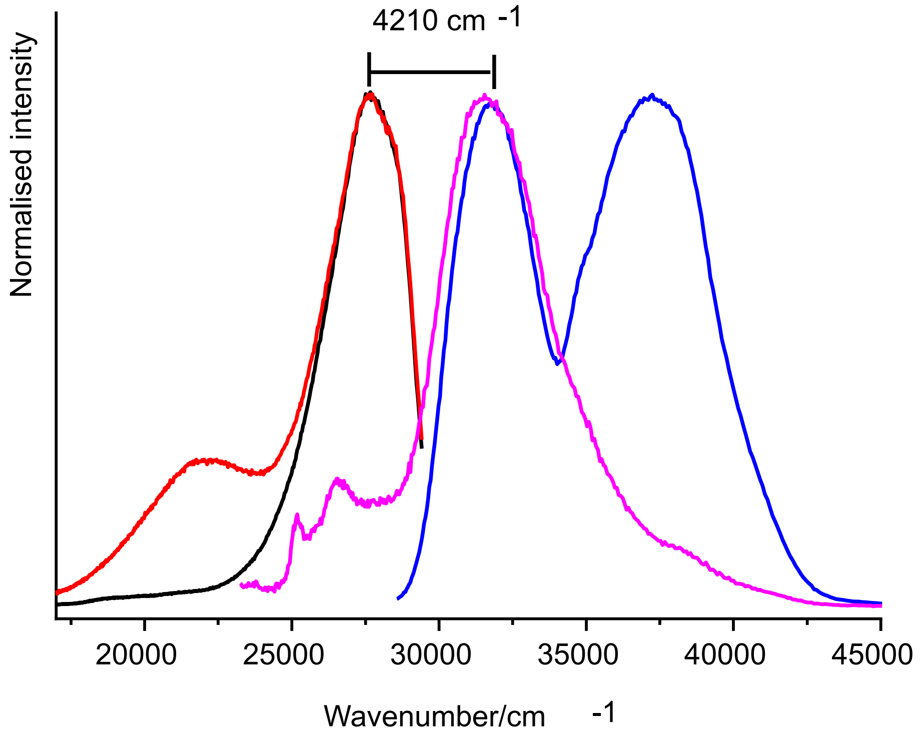

Figure S2 Calculation of the Stokes shift as 4210 cm^{-1}

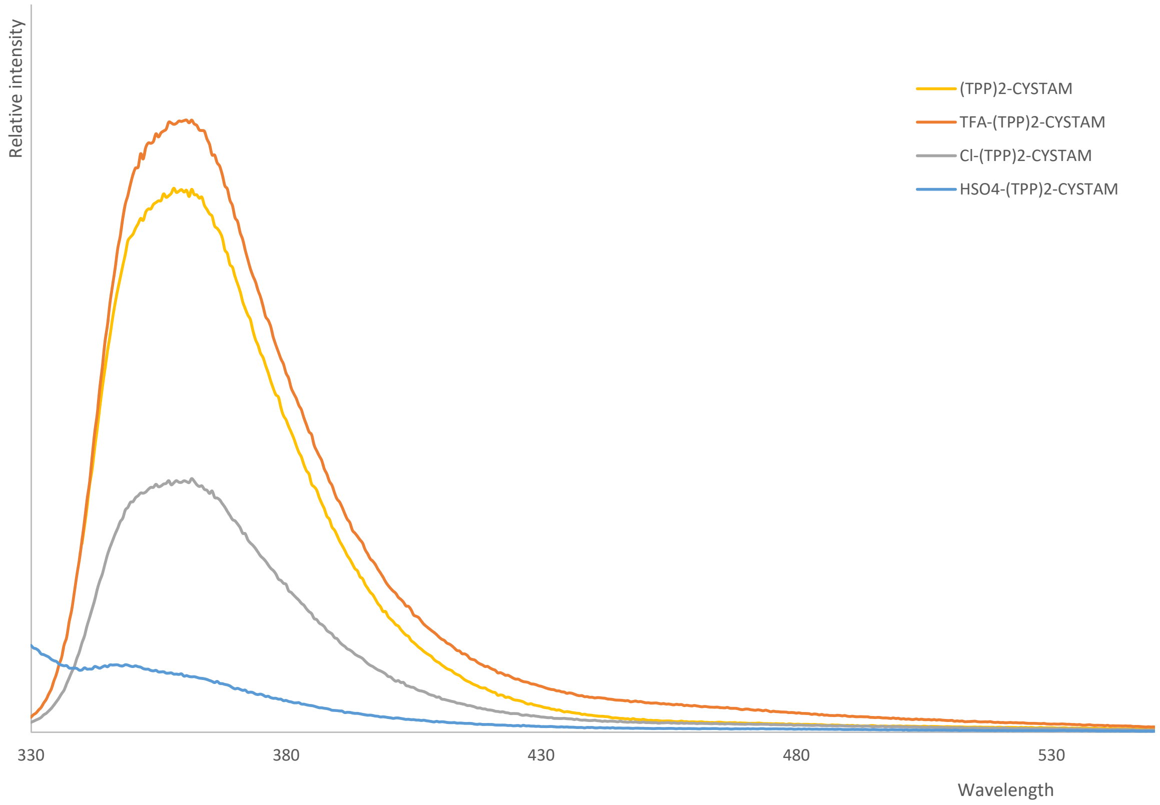

Figure S3 Emission spectra of of (TPP)2-CYSTAM and its adduct with TFA^-, Cl^-, HSO_4^-. Concentration of (TPP)2-CYSTAM = $1.37{\times}10^{-5}$ M. For each sample λ_{exc}=266 nm.
